# Phase Formation, Microstructure, and Magnetic Properties of Nd_14.5_Fe_79.3_B_6.2_ Melt-Spun Ribbons with Different Ce and Y Substitutions

**DOI:** 10.3390/ma14143992

**Published:** 2021-07-16

**Authors:** Qingjin Ke, Feilong Dai, Shengxi Li, Maohua Rong, Qingrong Yao, Jiang Wang

**Affiliations:** 1 Guangxi Key Laboratory of Information Materials, School of Materials Science and Engineering, Guilin University of Electronic Technology, Guilin 541004, China; keqingjin2021@163.com (Q.K.); dfldeyouxiang123@163.com (F.D.); lsx1027297604@163.com (S.L.); qingry96@guet.edu.cn (Q.Y.); 2 Engineering Research Center of Electronic Information Materials and Devices, Ministry of Education, Guilin University of Electronic Technology, Guilin 541004, China

**Keywords:** Nd-Ce-Y-Fe-B, melt-spun ribbon, phase structure, magnetic properties

## Abstract

Phase formation and microstructure of (Nd_1-2x_Ce_x_Y_x_)_14.5_Fe_79.3_B_6.2_ (x = 0.05, 0.10, 0.15, 0.20, 0.25) alloys were studied experimentally. The results reveal that (Nd_1-2x_Ce_x_Y_x_)_14.5_Fe_79.3_B_6.2_ annealed alloys show (NdCeY)_2_Fe_14_B phase with the tetragonal Nd_2_Fe_14_B-typed structure (space group P4_2_/mnm) and rich-RE (α-Nd) phase, while (Nd_1-2x_Ce_x_Y_x_)_14.5_Fe_79.3_B_6.2_ ribbons prepared by melt-spun technology are composed of (NdCeY)_2_Fe_14_B phase, α-Nd phase and α-Fe phase, except for the ribbon with x = 0.25, which consists of additional CeFe_2_ phase. On the other hand, magnetic properties of (Nd_1-2x_Ce_x_Y_x_)_14.5_Fe_79.3_B_6.2_ melt-spun ribbons were measured by a vibrating sample magnetometer (VSM). The measured results show that the remanence (B_r_) and the coercivity (H_cj_) of the melt-spun ribbons decrease with the increase of Ce and Y substitutions, while the maximum magnetic energy product ((BH)_max_) of the ribbons decreases and then increases. The tendency of magnetic properties of the ribbons could result from the co-substitution of Ce and Y for Nd in Nd_2_Fe_14_B phase and different phase constitutions. It was found that the H_cj_ of the ribbon with x = 0.20 is relatively high to be 9.01 kOe, while the (BH)_max_ of the ribbon with x = 0.25 still reaches to be 9.06 MGOe. It suggests that magnetic properties of Nd-Fe-B ribbons with Ce and Y co-substitution could be tunable through alloy composition and phase formation to fabricate novel Nd-Fe-B magnets with low costs and high performance.

## 1. Introduction

Since discovered in 1983, Nd-Fe-B magnets as outstanding magnetic materials have been used intensively in variously industrial applications such as motors of electric/hybrid vehicles and electric generators [1,2,3]. In general, Nd-Fe-B magnets would improve greatly their coercivity through the addition of the low-abundant and expensive rare earth (RE) metals Dy and Tb. With the emerging applications for the purpose of the electronic industry, the demand for Nd-Fe-B magnets is increasing rapidly, which causes the excessive use of rare earth metals Nd, Pr, Dy, and Tb. Meanwhile, the high-abundant and cheap rare earth metals La, Ce, and Y were not used effectively in the production of Nd-Fe-B magnets. Therefore, the integrated utilization of rare earth metals in Nd-Fe-B magnets is a promising way to achieve the sustainable development of Nd-Fe-B magnets [4,5,6,7,8,9,10,11,12,13,14,15,16,17,18,19,20,21,22,23,24,25,26,27,28,29,30,31,32,33,34]. However, the magnetic properties of Nd-Fe-B magnets with La, Ce, and Y have deteriorated unavoidably through the traditional fabrication technology because intrinsic magnetic properties of La_2_Fe_14_B, Ce_2_Fe_14_B, and Y_2_Fe_14_B phases are inferior to those of Nd_2_Fe_14_B, Dy_2_Fe_14_B, and Tb_2_Fe_14_B phases [35]. Recently, different fabrication technologies (e.g., double main phase method, dual alloy method, and grain boundary diffusion method) and microstructure optimization (e.g., grain boundary restructure) were developed to improve magnetic properties of Nd-Fe-B magnets with La, Ce, and Y [6,7,8,9,10,11,12,13,14,15,16,17,18,19,20,21,22,23,24,25,26,27,28,29,30,31,32,33,34].

In particular, the magnetic performance of Y and/or Ce substituted Nd-Fe-B alloys were investigated in the literature [26,27,28,29,30,31,32,33,34]. Chen et al. [26] reported that Nd_9-x_Y_x_Fe_72_Ti_2_Zr_2_B_15_ nanocomposite ribbons exhibited high coercivity (923.4 kA/m). Fan et al. [30] found that Y substituted Nd-Ce-Fe-B sintered magnets show special core–shell microstructure characteristics with Y-rich core and Nd-rich shell, resulting in the improvement of the coercivity and the thermal stability of magnets. Zheng et al. [32] studied magnetic properties of (Nd_1-x_Y_x_)_14.5_Fe_bal_B_6_Co_0.2_Al_1_Cu_0.15_ melt-spun ribbons. Zhang et al. [34] investigated phase constituent, microstructure, and magnetic properties of Nd_12-x_Y_x_Fe_81_B_6_Nb melt-spun ribbons. The results [32,34] show that the magnetic properties of ribbons reduce gradually with increasing Y content. Liao et al. [33] studied the effect of element distribution on the magnetic performance of (PrNd)-(Y_10-x_Ce_x_)-Fe-B sintered magnets. The remarkable increase of the coercivity of magnets with x = 2 indicated that Y-Ce co-substitution could improve the magnetic properties of Nd-Fe-B magnets. The experimental results [26,27,28,29,30,31,32,33,34] indicate the significant effect of alloy composition, microstructure, and fabrication technology on the magnetic properties of Nd-Fe-B alloys with Ce and/or Y substitution. In order to understand further the effect of phase formation and microstructure on magnetic properties of Nd-Fe-B alloys with Ce and Y co-substitution, phase structure, microstructure, and magnetic properties of (Nd_1-2x_Ce_x_Y_x_)_14.5_Fe_79.3_B_6.2_ alloys were studied experimentally in this work.

## 2. Experimental Procedure

Pure bulk Nd, Ce, Fe and B (99.99% purity) were used to prepare (Nd_1-2x_Ce_x_Y_x_)_14.5_Fe_79.3_B_6.2_ (x = 0.05, 0.10, 0.15, 0.20, 0.25) alloys by arc-melting method. After being melted four times, alloy samples were sealed in vacuum quartz tubes to be annealed at 1173 K for 360 h. The ribbons were obtained by induction melting the annealed alloys and then ejecting melts through the orifice (orifice diameter about 0.8–1.0 mm) of quartz tubes onto the copper wheel surface with wheel speeds of 20–30 m/s.

The crystal structures of the formed phases in the annealed alloys and melt-spun ribbons were examined using X-ray powder diffraction (XRD, PLXcel 3D, PANalytical, Almelo, the Netherlands, Cu K_α_ radiation). The microstructure of the annealed alloys was tested by scanning electron microscopy (SEM, Quanta FEG-450, FEI, Hillsboro, OR, USA). Magnetic measurements of the ribbons were carried out using the Lakeshore VSM (Model 7400 740H, Lake Shore Cryotronics, Westerville, OH, USA) at room temperature. The demagnetization correction of the ribbons was not considered because the applied magnetic field is parallel to the plane of the ribbons during the magnetic measurements.

## 3. Results and Discussion

### 3.1. Phase Structure and Microstructure

**Figure 1:**
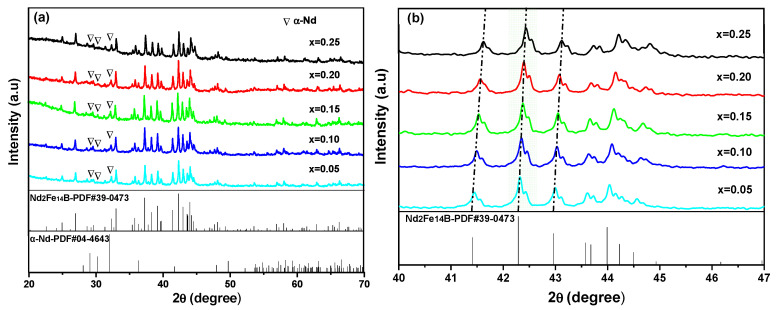
XRD powder patterns (**a**) and local XRD patterns (**b**) of (Nd_1-2x_Ce_x_Y_x_)_14.5_Fe_79.3_B_6.2_ alloys annealed at 1173 K for 360 h.

Figure 1 shows the XRD patterns of (Nd_1-2x_Ce_x_Y_x_)_14.5_Fe_79.3_B_6.2_ alloys annealed at 1173 K for 360 h. In Figure 1a, all alloys contain the (NdCeY)_2_Fe_14_B phase with isotropic Nd_2_Fe_14_B structure (space group P4_2_/mnm) and rich-RE (α-Nd) phase. Figure 1b presents the local XRD patterns of (Nd_1-2x_Ce_x_Y_x_)_14.5_Fe_79.3_B_6.2_ annealed alloys. The diffraction peak around 30° is belonged to the rich-RE (α-Nd) phase, while the weak diffraction peak at 45.4° confirms that the α-Fe phase was not formed. It is evident in Figure 1b that the diffraction peaks of the (NdCeY)_2_Fe_14_B phase shift slightly to the direction of high angle with the increase of Ce and Y substitutions because the lattice constants of Y_2_Fe_14_B (a = 0.8757 nm, c = 1.2026 nm) and Ce_2_Fe_14_B (a = 0.8726 nm, c = 1.2057 nm) are smaller than those of Nd_2_Fe_14_B (a = 0.8792 nm, c = 1.2177 nm) [35]. It suggests that Ce and Y atoms replace Nd atoms in the lattice of the Nd_2_Fe_14_B phase, leading to shifting diffraction peaks to the direction of high angles according to the Bragg equation [32].

**Figure 2:**
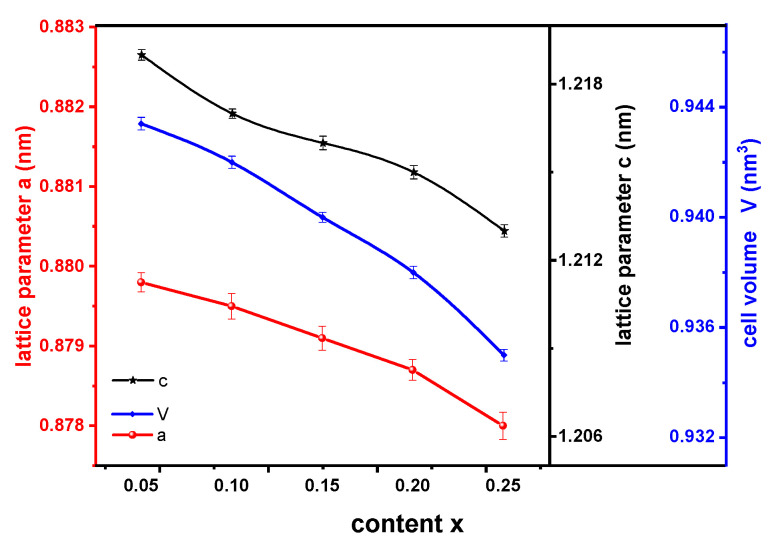
Lattice parameters and cell volumes of (NdCeY)_2_Fe_14_B phase in (Nd_1-2x_Ce_x_Y_x_)_14.5_Fe_79.3_B_6.2_ alloys annealed at 1173 K for 360 h.

Based on the crystal structure analysis, lattice parameters and cell volumes of (NdCeY)_2_Fe_14_B phase in annealed alloys were obtained, as shown in Figure 2. It can be seen that lattice parameters and cell volumes of (NdCeY)_2_Fe_14_B phase decrease slightly with the increase of Ce and Y substitutions in annealed alloys because of the replacement of Nd by Ce and Y in the lattice of Nd_2_Fe_14_B phase. 

**Figure 3:**
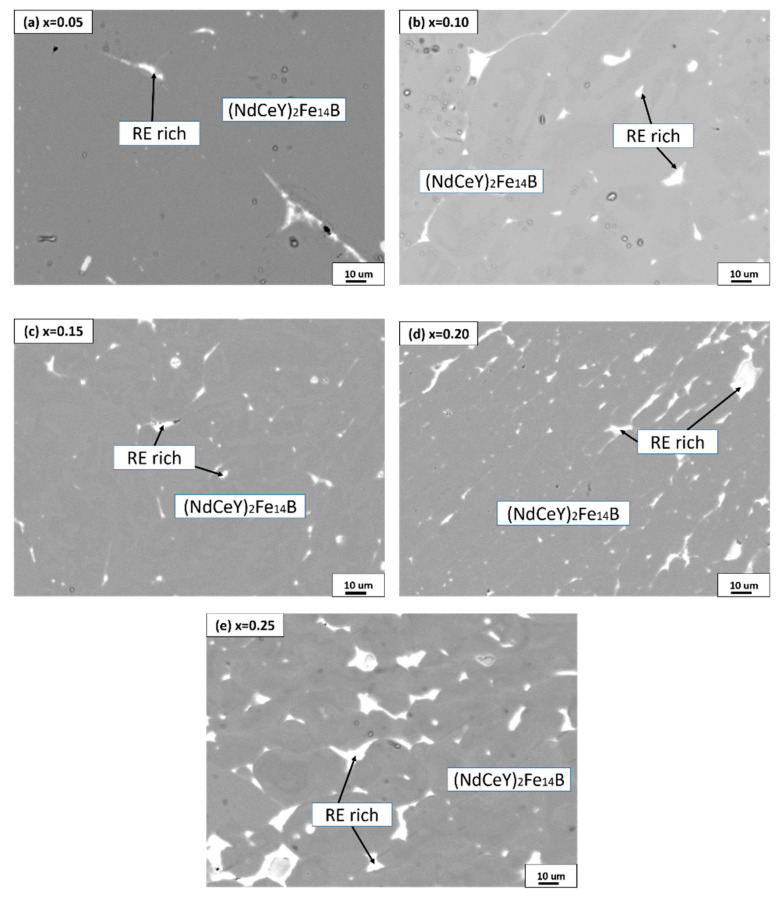
The backscattered electron (BSE) images of (Nd_1-2x_Ce_x_Y_x_)_14.5_Fe_79.3_B_6.2_ alloys annealed 1173 K for 360 h: (**a**) x = 0.05, (**b**) x = 0.10, (**c**) x = 0.15, (**d**) x = 0.20, and (**e**) x = 0.25.

Figure 3 is the microstructure image of (Nd_1-2x_Ce_x_Y_x_)_14.5_Fe_79.3_B_6.2_ annealed alloys. As can be seen, the microstructure characteristics of the annealed alloys show (NdCeY)_2_Fe_14_B phase (gray area) and rich-RE (α-Nd) phase (white area), while the α-Fe phase was not observed, which is in good agreement with the XRD results. It was found that the volume fraction of the rich-RE (α-Nd) phase (white area) in annealed alloys increases gradually with the increase of Ce and Y substitutions. 

**Figure 4:**
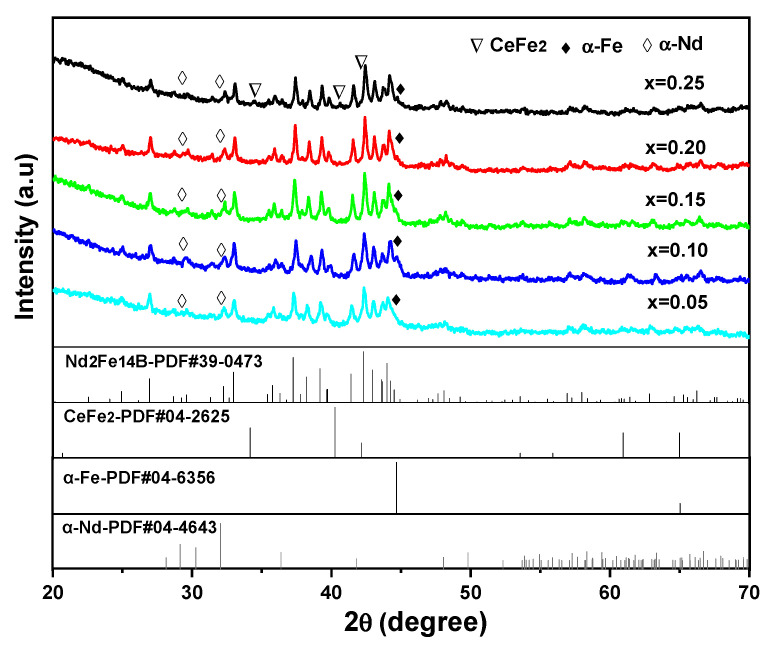
XRD patterns of (Nd_1-2x_Ce_x_Y_x_)_14.5_Fe_79.3_B_6.2_ (x = 0.05, 0.10, 0.15, 0.20, 0.25) melt-spun ribbons.

Figure 4 is the XRD patterns of (Nd_1-2x_Ce_x_Y_x_)_14.5_Fe_79.3_B_6.2_ melt-spun ribbons. According to the phase analysis of XRD patterns, diffraction peaks in all the ribbons are indexed as the (NdCeY)_2_Fe_14_B phase with the tetragonal Nd_2_Fe_14_B-typed structure (space group P4_2_/mnm), rich-RE (α-Nd) phase, and α-Fe phase. Especially, the ribbon with x = 0.25 has a different phase constitution, which contains additional CeFe_2_ phase.

### 3.2. Magnetic Properties

**Figure 5:**
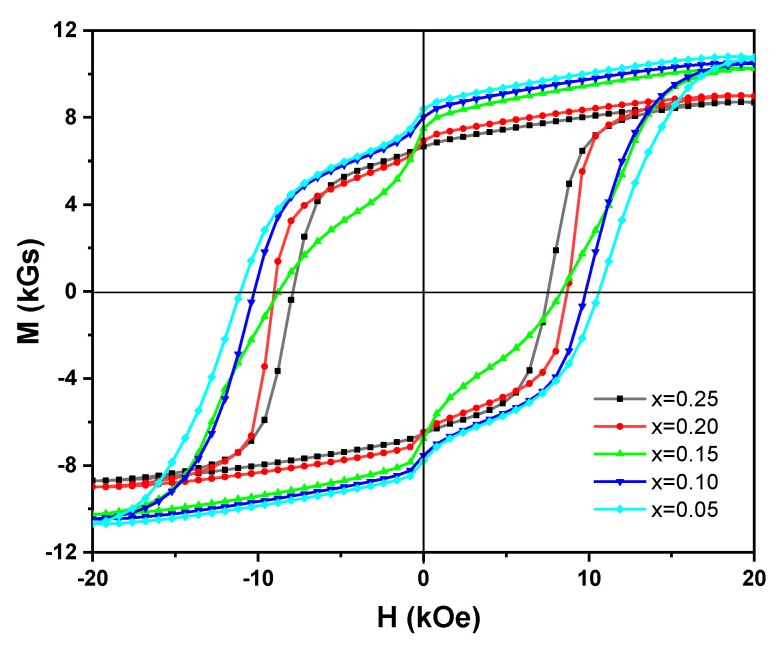
Hysteresis loops of (Nd_1-2x_Ce_x_Y_x_)_14.5_Fe_79.3_B_6.2_ (x = 0.05, 0.10, 0.15, 0.20, 0.25) melt-spun ribbons at room temperature.

Figure 5 is the hysteresis loops of (Nd_1-2x_Ce_x_Y_x_)_14.5_Fe_79.3_B_6.2_ melt-spun ribbons. It can be seen that the hysteresis loops show very poor squareness, which could result from the formation of α-Fe and CeFe_2_ phases in the ribbons. With the increase of Ce and Y substitutions, the remanent magnetization (M_r_) and saturation magnetization (M_s_) of the ribbons decrease monotonically because the intrinsic magnetic properties of Ce_2_Fe_14_B and Y_2_Fe_14_B phases are lower than those of the Nd_2_Fe_14_B phase [35].

Based on the hysteresis loops of (Nd_1-2x_Ce_x_Y_x_)_14.5_Fe_79.3_B_6.2_ melt-spun ribbons in Figure 5, the remanence (B_r_) and the coercivity (H_cj_) of the ribbons were obtained, while the maximum magnetic energy product ((BH)_max_) was determined from the area of the largest rectangle in the second quadrant of B-H curves transformed from the hysteresis loops. Table 1 summarizes magnetic properties of (Nd_1-2x_Ce_x_Y_x_)_14.5_Fe_79.3_B_6.2_ melt-spun ribbons. The remanence (B_r_) of (Nd_1-2x_Ce_x_Y_x_)_14.5_Fe_79.3_B_6.2_ melt-spun ribbons are 8.69 kGs, 7.72 kGs, 7.49 kGs, 6.65 kGs, and 6.64 kGs, respectively, while the coercivity (H_cj_) of the ribbons are 11.04 kOe, 10.21 kOe, 8.75 kOe, 9.01 kOe, and 7.85 kOe, respectively. In general, the remanence (B_r_) and the coercivity (H_cj_) of the ribbons deteriorate gradually with the increase of Ce and Y substitutions, except for the ribbon with x = 0.15, in which the coercivity (H_cj_) is higher than that of the ribbon with x = 0.10. The reason for it could result from the inferior magnetic properties of Ce_2_Fe_14_B and Y_2_Fe_14_B phases [35]. Based on the experimental results of the remanence (B_r_) and the coercivity (H_cj_) mentioned above, the maximum magnetic energy product ((BH)_max_) of the ribbons was determined to be 12.98 MGOe, 10.64 MGOe, 6.86 MGOe, 8.33 MGOe, and 9.06 MGOe, respectively. It was found that (BH)_max_ of the ribbon with x = 0.15 is the smallest due to the poor squareness of the hysteresis loop, while the ribbon with x = 0.25 has good magnetic performance (B_r_ = 6.64 kGs, H_cj_ = 7.85 kOe, (BH)_max_ = 9.06 MGOe), indicating that magnetic properties of Nd-Ce-Y-Fe-B ribbons could be regulated by alloy composition and phase formation. 

**Figure 6:**
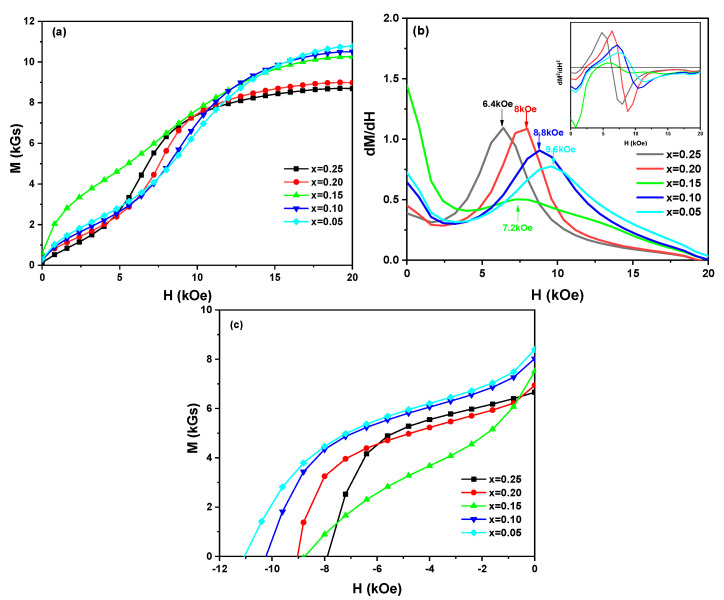
Initial magnetization curves (**a**), the first and second derivatives of the initial magnetization curve (**b**), and demagnetization curves (**c**) of (Nd_1-2x_Ce_x_Y_x_)_14.5_Fe_79.3_B_6.2_ (x = 0.05, 0.10, 0.15, 0.20, 0.25) melt-spun ribbons at room temperature.

Figure 6 shows the initial magnetization curves and demagnetization curves of (Nd_1-2x_Ce_x_Y_x_)_14.5_Fe_79.3_B_6.2_ melt-spun ribbons. From the shape of the initial magnetization curves in Figure 6a, the magnetization process of the ribbons is staged, and all ribbons are controlled by the nucleation model and the domain wall pinning model. The first and second derivatives of the initial magnetization curves of the ribbons are shown in Figure 6b, which analyze better the pinning field distribution and the magnetization characteristics. As can be seen, the magnetization processes of the ribbons with x = 0.05, 0.10, 0.20, 0.25 are mainly controlled by the domain wall pinning model, while the magnetization process of the ribbon with x = 0.15 is mainly controlled by the nucleation model. When the magnetic field is 0–2.5 kOe, the dM/dH along with the increase of magnetic field increases and declines rapidly, indicating that the magnetization process of the ribbons is dominated by nucleation at the beginning, which could be due to the existence of α-Fe phase in the ribbons. The α-Fe phase has exchange coupling with the (NdCeY)_2_Fe_14_B phase, which promotes the rapid magnetization of the primary phase. With the increase of the magnetic field, the presence of rich-RE phases provides a pinning field in (Nd_1-2x_Ce_x_Y_x_)_14.5_Fe_79.3_B_6.2_ ribbons during the magnetization process, and the magnetization process is mainly dominated by the domain wall pinning. The magnetization of the ribbons would overcome the nailing action of grain boundary to its domain wall. After reaching the critical magnetic field, the magnetization increases sharply to saturation magnetization. The second derivative of the initial magnetization curve has only one extreme point, which indicates that the distribution of the pinning field is relatively uniform. In addition, since the magnetocrystalline anisotropy constants of Ce_2_Fe_14_B and Y_2_Fe_14_B are lower than that of Nd_2_Fe_14_B [35], the strength of the pinning field decreases gradually with the increase of Ce and Y substitutions.

As shown in Figure 6c, the kinks were observed in the demagnetization curves of the ribbons (except for the ribbon with x = 0.25). This phenomenon could result from the formation and uniform distribution of the soft magnetic phase (such as α-Fe and CeFe_2_ phases) in the ribbons. In addition, the region of α-Fe and/or CeFe_2_ phase would partially exchange-couple to the adjacent (NdCeY)_2_Fe_14_B phase, reversing incongruously to present kinks in the demagnetization curves.

## 4. Conclusions

In this work, phase structure, microstructure, and magnetic properties of (Nd_1-2x_Ce_x_Y_x_)_14.5_Fe_79.3_B_6.2_ (x = 0.05, 0.10, 0.15, 0.20, 0.25) alloys were investigated. The following results could be obtained:

(1) The XRD and SEM results show that (Nd_1-2x_Ce_x_Y_x_)_14.5_Fe_79.3_B_6.2_ annealed alloys contain the (NdCeY)_2_Fe_14_B phase with the tetragonal Nd_2_Fe_14_B-typed structure (space group P4_2_/mnm) and rich-RE (α-Nd) phase. Meanwhile, (Nd_1-2x_Ce_x_Y_x_)_14.5_Fe_79.3_B_6.2_ melt-spun ribbons are composed of (NdCeY)_2_Fe_14_B phase, α-Nd phase and α-Fe phase, except for the ribbon with x = 0.25, which consists of additional CeFe_2_ phase.
(2) Magnetic measurements show that the B_r_ and the H_cj_ of (Nd_1-2x_Ce_x_Y_x_)_14.5_Fe_79.3_B_6.2_ ribbons decrease with the increase of Ce and Y substitutions, while the (BH)_max_ of the ribbons decrease and then increase. The tendency of magnetic properties of the ribbons could result from the co-substitution of Ce and Y for Nd in Nd_2_Fe_14_B phase and different phase formation.
(3) The H_cj_ of the ribbon with x = 0.20 is relatively high to be 9.01 kOe, while the (BH)_max_ of the ribbon with x = 0.25 is still 9.06 MGOe. It indicates that the magnetic performance of Nd-Ce-Y-Fe-B melt-spun ribbons would be regulated through alloy composition and phase formation to fabricate novel Nd-Fe-B magnets with low costs and high performance.

## Figures and Tables

**Table 1:** Magnetic properties of (Nd_1-2x_Ce_x_Y_x_)_14.5_Fe_79.3_B_6.2_ melt-spun ribbons.

Melt-Spun Ribbons (Nd_1-2x_Ce_x_Y_x_)_14.5_Fe_79.3_B_6.2_	B_r_ (kGs)	H_cj_ (kOe)	(BH)_max_ (MGOe)	M_r_ (emu/g)	Remanent Ratio (M_r_/M_s_)	Squareness
x = 0.05	8.69	11.04	12.98	83.71	0.78	0.63
x = 0.10	7.72	10.20	10.64	80.06	0.75	0.67
x = 0.15	7.49	8.75	6.86	74.83	0.73	0.46
x = 0.20	6.65	9.01	8.33	69.27	0.77	0.70
x = 0.25	6.64	7.85	9.06	66.43	0.76	0.76

## Data Availability

Data sharing is not applicable to this article.
